# Writing to learn on the wards: scholarly blog posts by medical students and housestaff at a teaching hospital

**DOI:** 10.1080/10872981.2018.1565044

**Published:** 2019-01-29

**Authors:** Farrin A. Manian, Felicia Hsu

**Affiliations:** a Core Educator Faculty, Department of Medicine, Massachusetts General Hospital, Boston, MA, USA; b Harvard Medical School, Boston, MA, USA

**Keywords:** Writing, housestaff, medical students, blog, post, medical education

## Abstract

**Background**: Informative writing is a valuable tool for learning and fostering the scientific process. Pearls4Peers (P4P) is an educational open-access website dedicated to scholarly blog posts in hospital medicine based on questions raised during ward teaching rounds. A goal of P4P is to enhance the learning experience of medical students and housestaff (i.e., interns and upper-level residents) by inviting them to write blog posts for a worldwide audience.

**Objective**: To describe our experience with inviting medical students and housestaff to contribute blog posts to a clinically oriented educational website with the aim of promoting concise evidence-based informative medical writing.

**Design**: Medical students and housestaff assigned to the hospital ward team of an attending physician (FM) on the medical service were routinely invited to submit one or more posts or ‘pearls’ based on clinical questions raised during patient rounds. Selected features of submissions during the first 2 years of P4P (27 June 2015 through 26 June 2017) were then retrospectively reviewed and analyzed.

**Results**: Of 156 pearls posted during the study period, 25 (16%) were contributed by medical students and 16 (10.3%) by housestaff. Medical students were significantly more likely to contribute than housestaff (19[70.4%] vs. 11 (9.6%], *p* < 0.01). Superfluous information was noted in 12 (29.3%) submissions. Word count exceeded the suggested limit of 200 words in 12 (29.3%) cases. An inverted pyramid structure, a widely recognized web writing format with the most important information presented at the outset, was noted in only 17 (41.5%) of entries. Unsolicited comments by contributors suggested a positive learning experience in writing the posts.

**Conclusions**: Writing clinically oriented concise blog posts appears feasible and may be an effective tool in enhancing the ward-based learning experience of medical students and housestaff. More formal instructions on the proper content and structure of blog posts seem warranted.

## Introduction

Informative or explanatory writing, defined as writing that explains and provides the reader with information rooted in facts about a topic or subject, is a valuable tool for learning and fostering the scientific thought process [1]. Unfortunately, this important activity may not always be sufficiently stressed during pre- and post-graduate medical education years, possibly contributing to a lack of confidence in writing, writing-related anxiety and cognitive burden among trainees [1]. Even informal writing as a way of enhancing personal understanding appears to benefit students, including better performance on related exam questions [1,2]. We, hereby, describe our experience with inviting medical students and housestaff (i.e., interns and upper-level residents) to contribute to a clinically oriented educational blog through concise evidence-based informative writing in hopes of enhancing their learning experience on the wards.

## Material and methods

In June, 2015, Pearls4Peers (P4P, www.pearls4peers.com), an educational non-commercial open-access website dedicated to scholarly blog posts in hospital medicine was launched by one of the authors (FM), a specialist in infectious diseases and hospital medicine practicing at Massachusetts General Hospital (MGH), a teaching hospital in Boston. The original mission of this educational website was to provide clear, concise, and evidence-based answers to clinical questions raised during hospital rounds, usually requiring less than 1 min to read. A related goal of P4P was to encourage medical students and housestaff to engage in scholarly informative writing by submitting a blog post of their own.

Following the launch of P4P in 2015, one of the authors (FM) who regularly serves as an attending physician on the general medicine teaching wards at MGH, routinely invited medical students and housestaff on his team to write and submit one or more informative blog posts or ‘pearls’ based on clinical questions raised during patient rounds. Potential contributors were asked to ‘deep-dive’ into a specific aspect of a clinical topic, distill the relevant information, and provide a concise evidence-based explanation. They were further incentivized to write by offering them an opportunity to display their contribution before a broader worldwide audience on the internet. Special emphasis was placed on writing pearls based on questions raised by the authors themselves. The invitation to contribute a blog post was announced at the beginning of each ward rotation and periodically thereafter as needed. Although submission of pearls contemporaneous to the ward rotation was preferred, those written subsequently were also accepted.

Suggested format of each pearl consisted of 3 basic elements: a specific clinical question, a concise written response of usually no more than 150–200 words, and supportive literature citations (usually 3–5 references) involving peer-reviewed published articles. When necessary, reference to textbooks or other highly regarded popular resources (e.g., Centers for Disease Control and Prevention) were also deemed appropriate. Direct reference to an online summary resource (e.g., Uptodate) was not accepted. These guidelines were routinely discussed with medical students and housestaff in advance of their writing. All submissions were emailed to FM for further editing as needed before formal posting. Contribution of the individual authors was fully acknowledged in each post.

For data analysis, all submissions originally emailed by medical students or housestaff during the first 2 years of P4P (27 June 2015 through 26 June 2017) were retrospectively reviewed by FM and assessed for content (i.e., word count, adequacy of content related to the posed question, appropriateness of references, and the presence of superfluous information not directly related to the posed question). The structure of each submitted piece was also assessed utilizing an ‘inverted pyramid’ format as the standard for comparison. The latter is a popular writing structure long adopted by newspapers and well-suited for prompt retrieval of information from the web, particularly through mobile units such as smart phones [3]. It consists of providing the most relevant information at the beginning of each piece (i.e., on the top of the inverted pyramid), followed by less crucial information related to the stated question in descending order [4]. Formal review of this study by the MGH Institutional Review Board was waived.

Rates of participation in this writing activity were calculated based on the number of invited medical students and housestaff by their respective levels of training during the study period. InStat (GraphPad, La Jolla, CA) software was used for statistical analysis. Categorical data were compared using Fisher’s exact test, with *p* < 0.05 considered statistically significant.

## Results

During the study period, a total of 156 pearls were posted on P4P of which 41 (26.3%) were contributed by either medical students or housestaff: 25 (16%) by medical students and 16 (10.3%) by housestaff (7 interns and 9 upper-level residents). Twenty authors (14 medical students and 6 housestaff) contributed 1 pearl each, 9 (5 medical students and 4 housestaff) provided 2 pearls and 1 intern contributed 3 pearls during the study period. Of 27 invited medical students, 19 (70.4%) submitted at least 1 pearl, compared to 11 (9.6%) of 114 invited housestaff (*p* < 0.01, OR 0.045, 95% confidence interval 0.016–0.13).

**Figure 1.**
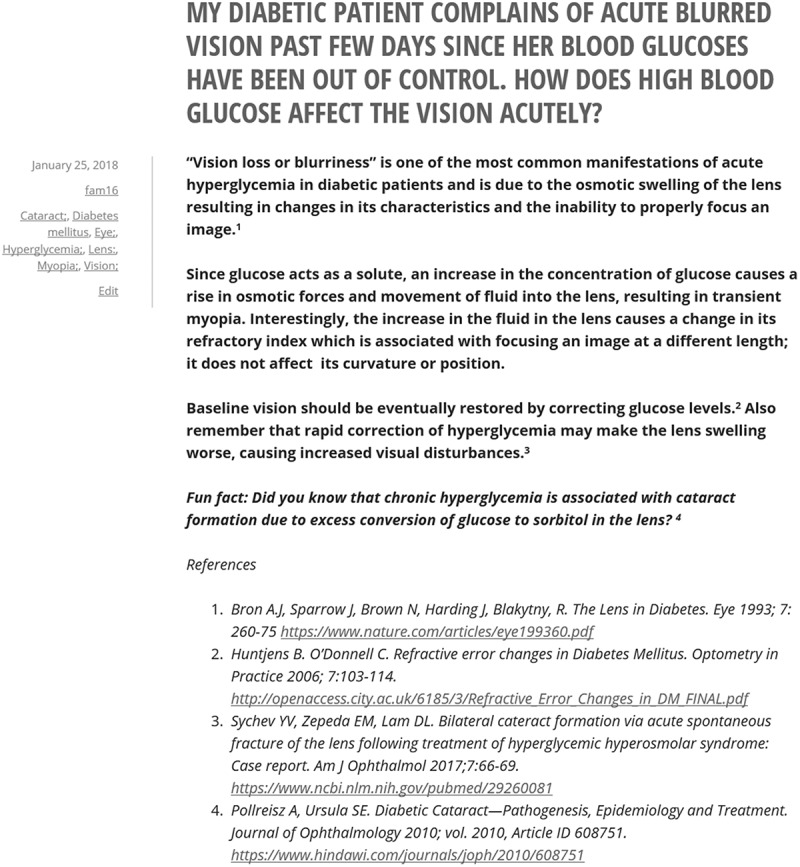
Actual pearl posted on Pearls4peers authored by a medical student based on visual changes in a diabetic patient with high blood glucoses.

**Figure 2.**
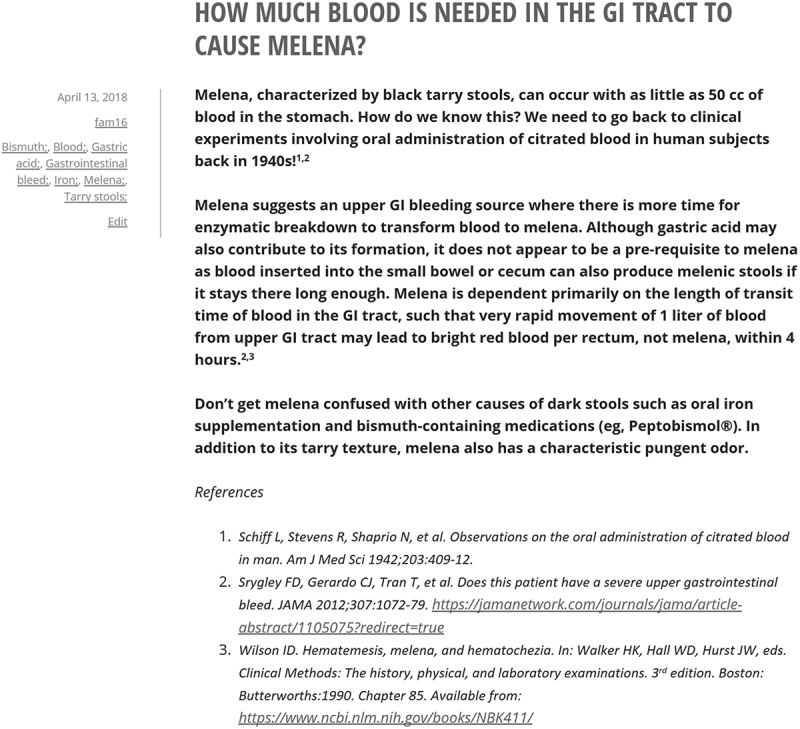
Actual pearl posted on Pearls4Peers authored by an upper-level resident based on the finding of melena in a patient with gastrointestinal bleeding.

The unedited submitted piece was considered to have adequate content in 35 (85.4%) cases, while superfluous information was noted in 12 (29.3%) entries. Word count ranged from 106 to 554 words (mean 210 words, median 178 words), with 12 (29.3%) submissions exceeding 200 words. Appropriate supportive citations were provided in 39 (95.1%) entries. An inverted pyramid structure in the presentation of posts could be found in 17 (41.5%) submissions. Comparison of pearls submitted by medical students with those of housestaff revealed no significant differences in the adequacy of content or citations, word count, or rates of superfluous information or adherence to an inverse pyramid format (data not shown). Ten sample questions serving the basis for a pearl authored by medical students and housestaff are shown (Table 1), and two actual posts on P4P are also displayed (Figures 1 and 2). As expected, a wide variety of clinical topics were covered by the pearls, reflecting the varied mix of patient ailments commonly observed on medical wards.

**Table 1.** Sample clinical questions serving as basis for writing respective pearls on Pearls4Peers.

Selected Questions	
1	My diabetic patient complains of acute blurred vision past few days since her blood glucoses have been out of control. How does high blood glucose affect the vision acutely?^a^
2	What is the significance of smudge cells on peripheral blood smear?^b^
3	How is prealbumin related to albumin?
4	Can native valve infective endocarditis be associated with hemolytic anemia?
5	Why are patients with acute exacerbation of COPD at higher risk of venous thromboembolism?
6	How much blood is needed in the GI tract to cause melena?
7	What is the utility of bedside skin-fold test in diagnosing Cushing’s syndrome?
8	Can my patient with cirrhosis and hepatocellular carcinoma still qualify for a liver transplant?
9	When should I seriously consider active tuberculosis (TB) in my newly admitted HIV-negative patient with a cough?
10	Which is more effective in managing rapid ventricular rate in atrial fibrillation? Diltiazem or metoprolol?

^a^ See Figure 1 for actual post.

^b^ See Figure 2 for actual post.

Informal feedback from contributors (either verbal, via email or based on comments posted on the P4P website) included: ‘I am used to writing scholarly research articles but this is tougher since I have to condense my thoughts and words.’; ‘Thanks for encouraging me to write something up on this topic! It was very educational…’; ‘I was struggling to whittle (the pearl) down, but you did it nicely!’; ‘I am really glad you managed to edit to 150 (words). I wasn’t able to’; ‘Love this! Extremely helpful for my internship…Thank you for this wonderful resource’.

## Discussion

Clinical teaching rounds in hospital wards serve as a fertile ground for learning by medical students and housestaff by nurturing the process of asking key clinical questions and problem-solving. They can also be used to encourage clear and concise scholarly writing. Aside from having ready context, scholarly writing in this setting is well aligned with the four principles of learning and memory or ‘The Four E’s of Effective Learning’, as suggested in teaching psychology: engaging interest, encoding important information, elaborating meaning of newly learned material and evaluating progress [5].

The process of writing a scholarly pearl as described begins with engaging interest in a specific topic raised during rounds and often related to an existing patient’s condition. Engagement is even more likely when a question is raised by the writer himself or herself. As for encoding important information, requiring potential authors to provide scientific evidence for their submissions necessitates a journey through the existing peer-reviewed literature before citing the most relevant published work. This process should be conducive to imparting and encoding broader knowledge beyond that required to answer the question at hand. The most enduring learning, however, results from the process of engaging in deeper thinking and elaboration of the meaning of newly learned material [5], which scholarly writing demands. Evaluating progress in learning in this setting may be performed by encouraging the medical students and housestaff to discuss their findings related to the pearl with the rest of the patient care team during subsequent ward rounds.

To the best of our knowledge, this is the first published work exploring the potential use of writing scholarly blog posts as a means of enhancing the learning experience of medical students and housestaff on medical wards. We found that while most medical students responded favorably to the invitation to contribute a pearl, only a minority of the invited housestaff participated in this activity (70.4% vs. 9.6%, respectively, *p* < 0.01). Several explanations may be offered for this finding, such as medical students likely having more time to deep dive into a topic during ward rotations. In addition, although submissions were purely voluntary, medical students might have been more likely to perceive this activity as a means of improving their overall performance during the rotation as viewed by the attending physician. Lack of confidence in informative writing might have also played a role in not contributing a pearl irrespective of the level of training. Indeed, as 1 of the medical students commented, the ability to write concisely and explain a concept in 200 words or less may in some ways be more challenging than writing a traditional research paper or review article. However, limiting the word count is useful in that it encourages distilling and organizing authors’ thoughts relevant to the question asked.

Although the great majority of submitted pearls contained adequate content and citations, nearly one-third exceeded the suggested 200 word limit with a similar number containing superfluous information not directly related to the posed question. As for structure, only 41.5% of submissions followed the inverse pyramid format designed to facilitate ready retrieval of information on the web. These findings suggest opportunities for curbing wordiness in informative writing and the need for increased familiarity with the inverse pyramid structure among medical students and housestaff. Adoption of proper writing skills as described need not be limited to scholarly blog posts as patient write-ups and explanatory emails may also benefit from further refinement in brevity and clarity.

Several limitations of the current work are worthy of emphasis. Our study involved only general medicine teaching wards within a single academic institution, potentially limiting the generalizability of its results to other specialties or institutions with academic programs dissimilar to ours. In addition, aside from discussing the word limit and the importance of providing appropriate supportive references, no formal instructions were provided to potential contributors in advance of writing a pearl. It is likely that the compliance with certain desirable features of submitted pieces (e.g., the inverted pyramid format) would have been higher had formal instructions been provided. These limitations notwithstanding, we believe the current findings provide a glimpse into the feasibility of the use of informative writing skills of medical students and housestaff as a tool to enhance their ward-based learning experience and also suggest opportunities for improvement in their writing skills.

In summary, clear and concise scholarly blog posts based on clinical questions raised during patient ward rounds as described lends itself well to the principles of effective learning by readily providing context, facilitating encoding of new knowledge in process and encouraging deeper thinking and reflection on relevant clinical topics among medical students and housestaff. Aside from the satisfaction of assimilating new knowledge, contributors can hone their skills in communicating through the written word and may be further ‘rewarded’ by sharing their work with a broader worldwide audience on the internet. Emphasizing ‘writing to learn’ through scholarly blogging as described may yet be another tool in enhancing the learning experience of medical students and housestaff while improving their communication skills. More formal instructions on the proper content and structure of blog posts seem warranted. Formal studies evaluating the role of writing scholarly blog posts as part of the medical education curriculum and its impact on learning are also needed.
